# Increased Iron Status during a Feeding Trial of Iron-Biofortified Beans Increases Physical Work Efficiency in Rwandan Women

**DOI:** 10.1093/jn/nxaa016

**Published:** 2020-01-31

**Authors:** Sarah V Luna, Laura M Pompano, Mercy Lung'aho, Jean Bosco Gahutu, Jere D Haas

**Affiliations:** 1 Division of Nutritional Sciences, Cornell University, Ithaca, NY, USA; 2 Centro Internacional de Agricultura Tropical-Uganda, Kawanda, Uganda; 3 School of Medicine and Pharmacy, College of Medicine and Health Sciences, University of Rwanda, Huye, Rwanda

**Keywords:** Rwanda, biofortification, iron deficiency, anemia, physical performance, energetic work efficiency

## Abstract

**Background:**

Iron-biofortified staple foods can improve iron status and resolve iron deficiency. However, whether improved iron status from iron biofortification can improve physical performance remains unclear.

**Objective:**

This study aimed to examine whether changes in iron status from an iron-biofortified bean intervention affect work efficiency.

**Methods:**

A total of 125 iron-depleted (ferritin <20 μg/L) female Rwandan university students (18–26 y) were selected from a larger sample randomly assigned to consume iron-biofortified beans (Fe-Bean; 86.1 mg Fe/kg) or conventional beans (control: 50.6 mg Fe/kg) twice daily for 18 wk (average of 314 g beans consumed/d). Blood biomarkers of iron status (primary outcome) and physical work efficiency (secondary outcome) were measured before and after the intervention. Work performed was assessed during 5-min steady-state periods at 0-, 25-, and 40-W workloads using a mechanically braked cycle ergometer. Work efficiency was calculated at 25 W and 40 W as the work accomplished divided by the energy expended at that workload above that expended at 0 W. General linear models were used to evaluate the relation between changes in iron status biomarkers and work efficiency.

**Results:**

The Fe-Bean intervention had significant positive effects on hemoglobin, serum ferritin, and body iron stores but did not affect work efficiency. However, 18-wk change in hemoglobin was positively related to work efficiency at 40 W in the full sample (*n* = 119; estimate: 0.24 g/L; 95% CI: 0.01, 0.48 g/L; *P* = 0.044) and among women who were anemic (hemoglobin <120 g/L) at baseline (*n* = 43; estimate: 0.64 g/L; 95% CI: 0.05, 1.23 g/L; *P* = 0.036). Among women who were nonanemic at baseline, change in serum ferritin was positively related to change in work efficiency at 40 W (*n* = 60; estimate: 0.50 μg/L; 95% CI: 0.06, 0.95 μg/L; *P* = 0.027).

**Conclusions:**

Increasing iron status during an iron-biofortified bean feeding trial improves work efficiency in iron-depleted, sedentary women. This trial was registered at clinicaltrials.gov as NCT01594359.

## Introduction

Iron deficiency is one of the most common micronutrient deficiencies worldwide, affecting women, children, and infants most severely. The most common consequence of iron deficiency is anemia, or a blood hemoglobin concentration below a specified level [<120 g/L for nonpregnant women of reproductive age (1)]. In Rwanda, anemia, which is used as an indicator of iron deficiency, affects 19% of women of reproductive age (2). Iron deficiency is prevalent among women of reproductive age because of the high demand for iron to compensate for menstrual losses. Functional consequences of iron deficiency include decreased physical performance and physical activity and fatigue (3).

Strategies to reduce the burden of nutritional iron deficiency include dietary diversification, supplementation, commercial food fortification, and biofortification. Of these 4 strategies, biofortification has the potential to become a sustainable, inexpensive, and effective solution at the population level, particularly in the rural poor who have little or no access to commercial fortification and supplementation interventions (4). In developing countries, diets of the rural poor contain high amounts of staple foods (such as beans, maize, wheat, and rice) but few micronutrient-rich foods such as fruits, vegetables, and animal and fish products. Biofortified crops—staple food crops that have been bred to contain higher amounts of micronutrients using conventional breeding—can increase the micronutrient content of the diet without changing dietary patterns. Consumption of beans in Rwanda is as high as 66 kg/y per capita (5); therefore, beans are a promising candidate for iron biofortification (6). The research reported here was conducted in the context of an efficacy trial of iron-biofortified beans to improve iron status in iron-deficient Rwandan women (7).

The efficacy of iron biofortification is generally examined by measuring changes in iron status biomarkers. Efficacy trials also provide an opportunity to test for the effect of changes in iron status on the functional consequences of iron deficiency, such as physical performance. Physical performance has been shown in laboratory studies to be compromised by iron deficiency and improved after the administration of therapeutic doses of supplemental iron (3, 8). Iron deficiency with and without anemia impacts several types of physical performance, including peak oxygen consumption, endurance, and energetic efficiency (9). Peak oxygen consumption is important for athletes or those performing high-intensity work, while endurance performance can be important for those needing to perform moderate physical activity for extended durations. However, for sedentary women, such as those involved in the present study, the most relevant measure of physical performance is work efficiency. Work efficiency reflects the amount of energy that is required to perform a given amount of work. This measure can be used for high-intensity exercise but can also reflect efficiency of performing activities of daily living, such as walking. Previous studies have shown that improving iron status through iron supplementation produces improvements in work efficiency in athletes (10, 11) as well as productivity among tea plantation workers and cotton-mill workers in various settings (12, 13).

The women in this study were primarily sedentary but did perform several low-intensity activities, such as walking, on a regular basis. As the women in this study were unlikely to regularly perform high-intensity exercise, a change in peak oxygen consumption or endurance performance would not be as relevant to their daily lives as work efficiency. Therefore, the objective of this analysis was to determine whether consumption of iron-biofortified beans affected physical performance, as measured by work efficiency. The secondary objective was to determine whether improvements in iron status, regardless of treatment group, were related to improvements in work efficiency.

## Methods

### Study design

The Rwandan biofortified bean efficacy study was a randomized, controlled, double-blinded feeding trial that took place at the University of Rwanda at Huye from January to May 2013. Details of the study design have been reported previously by Haas et al. (7) and are briefly summarized here.

Biofortified beans and control beans were grown from certified seeds at the International Center for Tropical Agriculture campus in Cali, Colombia. The iron content of the biofortified beans and control beans was determined by inductively coupled plasma MS to be 86.1 mg/kg and 50.6 mg/kg, respectively (7). All participants were randomly assigned by ID number using a computer-generated random-number program. Participants were randomly assigned to 1 of 4 color groups, where 2 color groups were served iron-biofortified beans and the other 2 color groups were served control beans. Randomization was performed by an independent third party. Participants received meals in a buffet-style cafeteria daily for every lunch and dinner during the study. At each meal, participants were provided 175 g (wet weight) of cooked beans along with a vegetable, 2 starch-based sides, and a tomato-based sauce. All served food and postmeal waste was weighed by research staff. Beans were served and weighed in a bowl that was separate from other food types, allowing for measurement of bean consumption separate from total food consumption. Feeding occurred for 18 wk from 7 January to 15 May 2013. Participants, research staff, and data analysts were blinded to the assignments until after the primary study results were analyzed.

Baseline blood collection occurred during the first week of feeding to measure indicators of iron status and inflammation (primary outcome of parent study). Baseline physical performance data were collected over a period of 3 wk in January (secondary outcome of parent study). Endline assessment of work efficiency was performed between 2 and 18 May 2013, whereas endline blood sampling occurred 13–15 May 2013.

### Study participants

**FIGURE 1:**
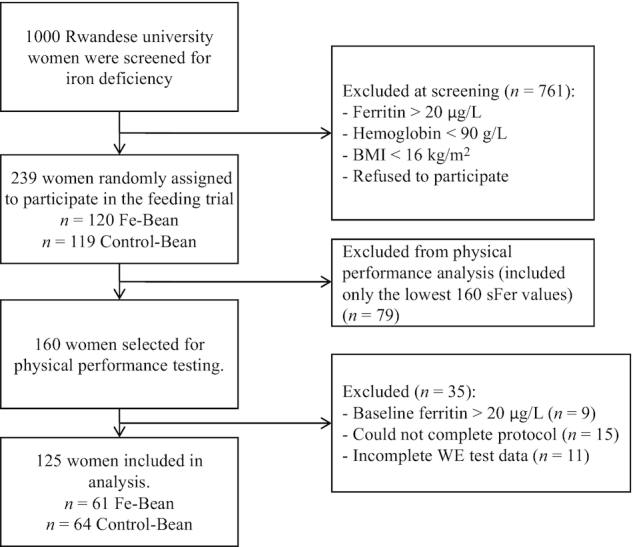
CONSORT diagram of sample selection. Control-Bean, treatment arm receiving control beans; Fe-Bean, treatment arm receiving iron-biofortified beans; sFer, serum ferritin; WE, work efficiency.TY

The study was conducted in iron-depleted but otherwise healthy young female students between the ages of 18 and 26 y in Huye, Rwanda. Inclusion and exclusion criteria for the intervention trial are described in the parent study (7). The flow of participants is shown in the CONSORT diagram in **Figure 1**. The 239 participants in the parent study were ranked by their serum ferritin (ferritin) values at screening, and the 160 women with the lowest ferritin values were selected to participate in the work efficiency testing. Potential participants were further excluded if they failed to complete a graded submaximal cycle ergometer test (*n* = 15), had baseline ferritin >20 μg/L (*n* = 9), or had incomplete data for the work efficiency test (*n* = 11). A ferritin cutoff of 20 μg/L was selected to match the cutoff used in the parent study, which used this cutoff because previous studies have shown that women with ferritin values above the clinical iron deficiency cutoff (15 μg/L) can still benefit in terms of their cognitive function and physical performance (9). The final sample size included in this analysis was 125 women (Figure 1).

Informed consent was obtained individually from the women participating in the study before the screening blood sample was taken. The Institutional Review Boards of Cornell University and the Rwanda National Ethics Committee approved the study protocols. The research permit was provided by the Rwanda Ministry of Education. This study was registered at clinicaltrials.gov (NCT01594359).

### Laboratory analyses

Whole-blood and serum collection procedures as well as iron and inflammation biomarker assays are described in detail in the parent study (7). Hemoglobin was assessed from whole blood using a Sysmex Automated Hematology Analyzer (model XS-1000i, Sysmex Corporation). Serum was analyzed for ferritin, C-reactive protein (CRP), soluble transferrin receptor (sTfR), and α1-acid glycoprotein (AGP) by sandwich ELISA (14). Laboratory serum samples were tested in batches by a senior technician, and instruments were calibrated daily based on standardized procedures.

Iron status was determined by concentrations of hemoglobin, ferritin, sTfR, and total body iron. A correction factor of 5 g/L was subtracted from all hemoglobin values based on the WHO recommendations for the altitude of Huye, Rwanda (15). Anemia was defined as hemoglobin <120 g/L. Iron deficiency was defined as a ferritin value <15 μg/L or an sTfR value >8.3 mg/L (16). Body iron stores were calculated for each subject from the sTfR to ferritin ratio using the equation reported by Cook and colleagues (17, 18), as follows:

(1) $$\begin{eqnarray*}
{\rm Body}\ {\rm iron}\,( {\rm mg/kg} )\,{=}\,{-}[{\rm log}\,( {\rm sTfR/ferritin}) - 2.8229]/0.1207
\end{eqnarray*}$$

Body iron values <0 mg Fe/kg body weight were considered iron deficient. Inflammation was assessed using CRP and AGP. Participants were categorized by inflammation status using previously published cutoffs (CRP >5 mg/L and AGP >1 g/L) (19).

### Assessing physical performance

In order to assess baseline fitness level and determine whether the participants had the skills to complete the work efficiency test, participants were instructed to complete a YMCA physical performance protocol on the cycle ergometer. The YMCA test is a graded submaximal exercise test that has been described previously (20). Participants were excluded from the study if they could not complete the YMCA test at baseline (*n* = 15). Reasons for not completing the test included reported fatigue, muscle soreness, shortness of breath, and inability to pedal at the required cadence. To reduce anxiety [elevated heart rate (HR) at rest], the women were acclimated to wearing the testing equipment and pedaling at the required cadence before beginning the exercise test protocol.

The primary outcome of this analysis was work efficiency, calculated by dividing the mechanical power performed on the ergometer (in kilocalories per minute converted from watts) by the energy expended (kilocalories per minute) at a given workload (25 W or 40 W) minus that expended during no-load cycling (0 W) (21, 22). Metabolic output was determined via indirect calorimetry by measuring gas exchange using a COSMED K_4_b^2^ system (COSMED K_4_b^2^; COSMED) during a work efficiency test performed on a mechanically braked and calibrated Monark 874E cycle ergometer (Vansbro). The work efficiency protocol is shown in the online supplemental materials (**Supplemental Table 1**). The protocol included three 4-min, low-intensity workloads (0 W, 25 W, and 40 W) separated by 3 min of rest to allow for estimation of work efficiency during an aerobic steady state achieved in the last 2 min of each workload. The reliability of the work efficiency itself was assessed using a test–retest protocol, which showed a technical error of measurement of 7%. Biologically impossible work efficiency values (<0% or >100%) were excluded from analysis (*n* = 16 and *n* = 12 for baseline and endline work efficiency at 25 W, respectively, and *n* = 2 and *n* = 1 for baseline and endline work efficiency at 40 W, respectively).

Metabolic parameters were assessed by the COSMED K_4_b^2^ portable metabolic measurement system, which analyzed the volume of respiratory air, concentrations of oxygen and carbon dioxide in expired air, and HR. Calculated metabolic variables included breath-by-breath measurements of oxygen consumption (*V*O_2_; liters per minute), exhaled carbon dioxide (*V*CO_2_; liters per minute), HR (beats per minute), and energy expenditure [kilocalories per minute using the Weir equation (23)].

### Statistical analyses

This study was designed to investigate the impact of consuming iron-biofortified beans compared with conventional beans on work efficiency. With the current sample size, we can detect the following differences between the iron-biofortified bean (Fe-Bean) and control (Control-Bean) groups at 90% power at a significance level (*P*) of 0.05: a 7.0-g/L difference in hemoglobin, a 2.1-μg/L difference in ferritin, a 2.3-mg/L difference in sTfR, a 1.4-mg/kg difference in body iron stores, and a 5.4% difference in work efficiency.

Descriptive statistics are reported as means ± SDs. Group differences for continuous variables were tested by using *t* tests or 1-factor ANOVA. Group differences for categorical binary variables were tested using Pearson's chi-square tests. Univariate general linear models were used to examine relations between inflammation (CRP or AGP) and ferritin.

Intent-to-treat (ITT) analyses were reported for all iron status markers and work efficiency (a secondary outcome of the larger trial). ITT analyses were run as general linear models of endline iron biomarker or work efficiency predicted by treatment group, controlling for the appropriate baseline value as well as baseline and endline CRP.

Secondary analyses were performed to assess the relation between changes in iron status and work efficiency using general linear models of endline work efficiency, controlling for work efficiency at baseline, baseline CRP, and endline CRP. Secondary analyses were also run with the same model structure but also including an interaction between the baseline to endline change in ferritin or hemoglobin and anemia status at baseline (*n* = 119). Finally, secondary analyses were run stratifying by baseline anemia status (*n* = 60 nonanemic and *n* = 43 anemic). Stratified analyses included reproducing the ITT and secondary models with the same covariates for anemics and nonanemics as well as comparing changes in iron status biomarkers between anemics and nonanemics (1-factor ANOVA). Multiple comparisons were adjusted for in all models (ITT, secondary, and stratified) using a Tukey correction. All effect estimates from linear models are presented as β estimates with 95% CIs and *P* values.

All data were analyzed using SAS version 9.4 (SAS Institute, Inc.). Statistical significance was set at ɑ = 0.05 for main effects and 0.10 for interaction effects.

## Results

A subsample of 125 Rwandan women were selected from the larger study sample to complete the physical performance tests (Figure 1). Among this subset, 38.4% were anemic, 84.8% had ferritin <15 μg/L, and 62.4% had negative body iron values at baseline. There were no differences between treatment groups for any baseline measure (**Table 1**). Both intervention groups consumed similar quantities of beans (mean = 38.4 kg wet weight) over the entire study period, or 157 g/meal. Since the beans differed in the amount of iron they contained, the women in the Fe-Bean group consumed significantly more iron per day from beans (14.7 mg/d) than the women in the Control-Bean group (9.3 mg/d) over the 18 wk of cafeteria feeding. Based on information on the fractional absorption of iron for the beans used in this study, as reported by Petry et al. (24), we computed the amount of absorbed iron for each subject. Assuming 7.2% absorption for the biofortified beans and 9.1% absorption for the control beans, the Fe-Bean group absorbed 1.04 mg Fe/d and the Control-Bean group absorbed 0.85 mg Fe/d. The Estimated Average Requirement (EAR) for US women of reproductive age is 1.46 mg Fe/d (25).

**TABLE 1:** Baseline characteristics of Rwandan women and bean consumption over 18 wk, by treatment group (Fe-Bean or Control-Bean)^1^

	Fe-Bean (*n* = 61)	Control-Bean (*n* = 64)
Subject biometrics		
Age, y	22.6 ± 1.5	22.8 ± 1.7
Weight, kg	57.2 ± 7.3	56.8 ± 7.6
Height, cm	160.0 ± 6.3	160.1 ± 6.3
e*V*O_2_max, mL · kg^−1^ · min^−1^	29.6 ± 4.9	31.0 ± 5.0
Work efficiency at 25 W, %	45.6 ± 15.8	45.2 ± 12.1
Work efficiency at 40 W, %	35.9 ± 10.4	36.7 ± 13.3
EnEx at 25 W, kcal/min	3.8 ± 0.5	3.8 ± 0.5
EnEx at 40 W kcal/min	4.7 ± 0.5	4.7 ± 0.6
Serum ferritin, μg/L	10.2 ± 5.4	10.7 ± 6.6
Hemoglobin,^2^ g/L	122.1 ± 13.7	122.5 ± 13.6
sTfR, mg/L	8.4 ± 3.9	8.1 ± 3.6
Body iron, mg/kg	−0.9 ± 2.8	−0.7 ± 2.9
CRP, mg/L	1.1 ± 1.4	0.8 ± 1.0
AGP, g/L	0.7 ± 0.2	0.7 ± 0.2
Bean consumption over 18 wk		
Total meals consumed	209 ± 24	206 ± 21
Total beans consumed, kg	38.4 ± 3.9	38.5 ± 3.7
Total iron consumed from beans, mg/18 wk	1649 ± 166*	963 ± 94
Total iron absorbed from beans, mg/18 wk	120 ± 12*	89 ± 9

1 Values are means ± SDs. *Indicates different from Control-Bean group based on a Student *t* test, *P* < 0.05. AGP, α1-acid glycoprotein; Control-Bean, treatment arm receiving control beans; CRP, C-reactive protein; EnEx, energetic expenditure; e*V*O_2_max, estimated maximal oxygen consumption; Fe-Bean, treatment arm receiving iron-biofortified beans; sTfR, soluble transferrin receptor.

2 Anemia defined as hemoglobin <120.0 g/L.

Inflammation (defined as CRP >5 mg/L or AGP >1 g/L) was present in 9.6% of the women. While overall prevalence of inflammation increased from baseline to endline (9.6% to 17.6%), there were no significant differences between treatment and control groups for endline CRP (Fe-Bean: 8.2%; Control-Bean: 6.3%; chi-square, *P* = 0.67) or AGP (Fe-Bean: 18.0%; Control-Bean: 12.5%; chi-square, *P* = 0.39). In addition, there were no differences between the Fe-Bean and Control-Bean groups for baseline to endline change in CRP (1.7 ± 5.4 mg/L vs 1.2 ± 4.3 mg/L, respectively; 1-factor ANOVA, *P* = 0.34) or AGP (0.1 ± 0.3 mg/L vs 0.1 ± 0.2 mg/L, respectively; 1-factor ANOVA, *P* = 0.90).

In univariate general linear models, baseline CRP significantly predicted baseline ferritin (β estimate: 0.94; 95% CI: 0.08, 1.81; *P* = 0.033). Baseline AGP did not significantly predict ferritin and neither CRP nor AGP predicted ferritin at endline (data not shown). Baseline to endline change in CRP predicted change in ferritin (β estimate: 0.23; 95% CI: −0.01, 0.47; *P* = 0.055). Baseline to endline change in AGP did not predict change in ferritin (β estimate: 3.95; 95% CI: −0.81, 8.50; *P* = 0.10). Therefore, only baseline and endline CRP values were included as covariates in all models. In order to assess the impact of inflammation in this analysis, all analyses were run excluding the 12 or 22 participants who had clinical inflammation based on either CRP >5 mg/L or AGP >1 mg/L at baseline or endline, respectively. Excluding these participants did not greatly change the effect size, direction, or significance of the results. Therefore, these participants were retained in all analyses.

### ITT analyses

Changes in iron status values over the course of the study have been reported previously using the full study sample (*n* = 239) (7). The key effects of the intervention on iron status for the subsample in the present analysis (**Table 2**) were similar to those reported in the parent study, finding significant treatment effects for baseline to endline change in hemoglobin and body iron. However, where the parent study reported only a trend towards a significant treatment effect for change in ferritin (*P* = 0.07; Table 4 in reference 7), the present study found that the Fe-Bean group had a significantly greater baseline to endline change in ferritin compared with the Control-Bean group (Table 2). There were no significant effects of the intervention on energy expenditure or work efficiency at 25 W or 40 W in the ITT analysis (**Table 3**).

**TABLE 2:** Endline iron status biomarkers in Rwandan women, by intervention group (Fe-Bean or Control-Bean)^1^

	Adjusted endline values after 18 wk		
	Fe-Bean (*n* = 61)	Control-Bean (*n* = 64)	Difference between groups
Hemoglobin, g/dL	12.5 (12.3, 12.7)	12.2 (12.0, 12.4)	0.3 (0.0, 0.5)*
Log ferritin, ln μg/L^2^	2.6 (2.5, 2.7)	2.4 (2.3, 2.5)	0.2 (0.0, 0.3)*
sTfR, mg/L	8.0 (7.5, 8.6)	8.1 (7.6, 8.7)	−0.1, (−0.9, 0.7)
Body iron, mg/kg	0.5 (0.1, 0.9)	−0.1 (−0.6, 0.3)	0.6 (0.0, 1.3)*

1 Values are least-square means (95% CIs), from general linear models adjusted for baseline value as well as baseline and endline CRP and adjusted for multiple comparisons using the Tukey adjustment method. *Indicates a *P* value <0.05. Control-Bean, intervention arm receiving conventional beans; CRP, C-reactive protein; Fe-Bean, intervention arm receiving iron-biofortified beans; sTfR, soluble transferrin receptor.

2 Ferritin was log transformed. Least-square means (95% CI) for the untransformed model (for easier interpretation of the effect) were as follows: Fe-Bean, 15.1 μg/L (13.6, 16.7); Control-Bean, 12.3 μg/L (10.8, 13.7); difference between groups, 2.9 μg/L (0.7, 5.0).

**TABLE 3:** Endline work efficiency of Rwandan women by treatment group (Fe-Bean or Control-Bean) or change in iron biomarker^1^

Outcome	Predictor	*n*	LS means (95% CI) for group	*P*
ITT analysis				
WE at 25 W	Treatment group	97	Fe-Bean: 48.2% (44.0%, 52.4%); Control: 47.2% (43.2%, 51.3%)	0.74
WE at 40 W	Treatment group	119	Fe-Bean: 37.0% (34.4%, 39.5%); Control: 36.6% (34.1%, 39.0%)	0.82
Secondary analyses				
Full sample (*n* = 125)				
Outcome				
WE at 25 W	Change Hb^2^	97	0.17 (−0.23, 0.58)^3^	0.41
WE at 25 W	Change Fer^4^	97	−0.29 (−0.75, 0.16)^3^	0.21
WE at 40 W	Change Hb^2^	119	0.24 (0.01, 0.48)^3^	0.044
WE at 40 W	Change Fer^4^	119	0.03 (−0.25, 0.32)^3^	0.83
IDNA at baseline (*n* = 60)				
Outcome				
WE at 40 W	Change Hb^2^	60	0.24 (−0.10, 0.58)^3^	0.16
WE at 40 W	Change Fer^4^	60	0.50 (0.06, 0.95)^3^	0.027
IDA at baseline (*n* = 43)				
Outcome				
WE at 40 W	Change Hb^2^	43	0.64 (0.05, 1.23)^3^	0.036
WE at 40 W	Change Fer^4^	43	0.10 (−0.76, 0.96)^3^	0.82

1 All models adjusted for baseline WE at 25 W or 40 W, as appropriate, baseline CRP, and endline CRP. Control-Bean, intervention arm receiving conventional beans; CRP, C-reactive protein; Fe-Bean, intervention arm receiving iron-biofortified beans; Fer, serum ferritin; Hb, hemoglobin; IDA, iron-depleted anemic; IDNA, iron-depleted nonanemic; ITT, intent-to-treat; LS, least square; WE, physical work efficiency.

2 Hb is reported in units of g/L.

3 Values are β estimates (95% CI).

4 Ferritin is reported in units of μg/L. Ferritin was not transformed for any secondary analysis because the residuals for the models were normally distributed in models using the untransformed variable but were not when using the log-transformed variable.

### Secondary analyses

Several secondary analyses were conducted examining changes in iron status and changes in work efficiency. Table 3 shows the results of all secondary analyses. There was a significant, positive relation between baseline to endline change in hemoglobin and endline work efficiency at 40 W in the total sample (*n* = 119) in a linear model accounting for baseline work efficiency, baseline CRP, and endline CRP (β estimate for change in hemoglobin: 0.24 g/L; 95% CI: 0.01, 0.48; *P* = 0.044). Change in hemoglobin was not significantly related to endline work efficiency at 25 W. No significant relations were observed between baseline to endline change in ferritin and endline work efficiency.

Because hemoglobin and ferritin have been shown to have differing effects on physical performance [hemoglobin affecting maximal oxygen uptake (9, 26), ferritin affecting endurance and work efficiency (26)], models were run with work efficiency as the dependent variable and included the interaction between baseline status (anemic or nonanemic) and baseline to endline change in ferritin or hemoglobin. A significant interaction was observed between baseline anemia status and baseline to endline change in ferritin as it affects work efficiency (interaction *P* = 0.087). Therefore, separate secondary analyses were run in women who were anemic (hemoglobin ≤120 g/L; *n* = 43) and nonanemic (hemoglobin >120 g/L; *n* = 60) at baseline (Table 3). For those women who were anemic at baseline, a similar result was observed as was seen in the full sample, with change in hemoglobin being positively related to endline work efficiency at 40 W, adjusted for baseline work efficiency, baseline CRP, and endline CRP (effect estimate: 0.64 g/L; 95% CI: 0.05, 1.23). For those women who were iron depleted but not anemic at baseline, change in hemoglobin was not significantly related to endline work efficiency at 40 W. However, baseline to endline change in ferritin was positively related to endline work efficiency at 40 W among women who were not anemic at baseline, adjusting for baseline work efficiency at 40 W, baseline CRP, and endline CRP (effect estimate: 0.50 μg/L; 95% CI: 0.06, 0.95).

Additionally, women who were anemic at baseline showed greater changes in baseline to endline hemoglobin than women who were nonanemic (3.27 g/L vs 0.21 g/L, respectively; 1-factor ANOVA, *P* = 0.019). In contrast, women who were nonanemic at baseline showed greater improvements in sTfR than women who were anemic at baseline (0.14 g/L vs −0.93 g/L, respectively; 1-factor ANOVA, *P* = 0.023). No differences were observed between anemic and nonanemic women for baseline to endline change in ferritin or body iron (data not shown).

## Discussion

This study suggests that the consumption of iron-biofortified beans produces significant improvements in iron status and that these changes in iron status affected physical performance (work efficiency) when exercising at moderate intensities. As a randomized, double-blinded, controlled efficacy trial, this study can contribute evidence for both the efficacy of biofortification as a dietary iron intervention and for the relation between improved iron status and improved work efficiency. The strong study design allowed for rigorous primary and secondary analyses. With 38.4% anemia and 84.8% iron deficiency at baseline, the potential to benefit from the intervention is high. Moreover, participants in the Fe-Bean group and the Control-Bean group were similar at baseline in terms of iron status and all measured potential confounding factors.

The intervention length was sufficient to allow for a detectable transfer of iron to body stores in the Fe-Bean group as reflected by significant increases in hemoglobin and ferritin. The hematological results of the larger Rwandan biofortification trial are reported elsewhere in more detail (7). The 160 participants with the lowest ferritin concentrations at screening were selected to participate in the physical performance testing. Therefore, the physical performance subsample analyzed in this article had a higher prevalence of both iron deficiency and anemia than the parent sample included in the biofortification efficacy trial, which likely explains why the present analysis observed significant intervention effects in hemoglobin, ferritin, and body iron, whereas the parent study observed only significant effects on ferritin (7).

The Fe-Bean and Control-Bean groups consumed similar amounts of beans throughout the trial. Both groups consumed sufficient amounts of iron through beans to exceed the EAR (8.1 mg/d) for iron for >50% of women of reproductive age (27), with the Fe-Bean group consuming significantly more iron from beans (14.3 mg/d) than the Control-Bean group (9.2 mg/d). Adjusting for the estimated fractional absorption of iron as reported by Petry et al. (24), the Fe-Bean group achieved 71% of their physiological requirement for iron from beans while the Control-Bean group achieved 58% of their requirement. Given that the diet consumed by the participants was exclusively vegetarian and had limited sources of additional dietary iron, many of the subjects were likely to have remained iron deficient, but the Fe-Bean group should have closed the gap to a greater extent than the Control-Bean group.

This study found that women who improved their hemoglobin were able to work at a greater level of work efficiency at a 40-W workload. This workload is equivalent to 2–3 metabolic equivalents and is considered to be “light” physical activity (28). Other forms of light physical activity include the following: picking fruits/vegetables, general cleaning and household activities, loading/unloading a car, and walking at a slow or “social” pace (28). Our accelerometer data indicate that subjects in the present study spend the majority of their time in sedentary behaviors or performing light physical activities, such as those listed above (data not shown). Therefore, an improvement in work efficiency in this intensity range should be relevant for the activities commonly performed by this group of university women.

It is likely that no treatment effect was observed in the ITT analysis because women in both the Fe-Bean and the Control-Bean groups consumed enough iron to produce meaningful improvements in their hemoglobin concentrations. Therefore, the relation between change in hemoglobin concentration and endline work efficiency at 40 W was not restricted only to those women who consumed the high-iron beans.

When examining the full subsample used in the present study (*n* = 125), the improvements in work efficiency were related to changes in hemoglobin rather than ferritin. Previous literature has supported that improving hemoglobin directly increases peak oxygen consumption but has a weaker relation with energetic efficiency (9). This is primarily because the body's capacity for delivering oxygen to exercising muscles increases directly with hemoglobin concentration, while work efficiency is generally related more to mitochondrial function (9, 10, 29). The existing studies examining energetic efficiency in iron-deficient populations have studied iron-deficient, nonanemic women, because the effects of anemia can mask smaller changes in work efficiency from iron deficiency alone (9–11, 30).

For this reason, we also conducted separate analyses examining the relation between change in iron status and change in work efficiency based on participants’ anemia status at baseline (anemic or not anemic). Indeed, when stratified by baseline status, women who were anemic at baseline showed improvements in work efficiency as their hemoglobin (but not ferritin) concentrations increased, while improvements in work efficiency in women who were not anemic at baseline were related only to changes in ferritin. The relation between ferritin and work efficiency in the iron depleted, nonanemic women in this study supports numerous other studies in similar populations (9, 26). However, the present study also included anemic women. It is likely that these women first repleted their hemoglobin, as evidenced by the larger increase in baseline to endline hemoglobin in the women who were anemic at baseline compared with those who were not. An improvement in hemoglobin would be expected to improve maximal oxygen consumption (not measured); however, their overall improvements in iron status may have also produced changes in work efficiency via increased iron availability for iron-dependent bioenergetics pathways in the mitochondria that would not be captured by the iron biomarkers measured in this study (9, 26).

The current study had several limitations. First, physical performance was a secondary measure; therefore, the study may not be powered to detect small changes in performance due to the intervention. We were not able to directly demonstrate a causal link between biofortified bean consumption and improvements in work efficiency in the present study, either in the ITT analysis or the secondary analyses. The current study involved low doses of iron in a biofortified staple food crop consumed by untrained women rather than physically active women, so it may not be reasonable to expect the same magnitude of treatment effect as seen in the previously mentioned reviews or in the Pasricha et al. (8) meta-analysis. In a placebo-controlled, iron supplementation trial, Zhu and Haas (30) showed the effect of improved iron status on *V*O_2_max and δ-efficiency (a measure of work efficiency) on nominally healthy, iron-depleted women who were untrained but physically active. Finally, this study was conducted under highly controlled experimental conditions. The study design may thus limit the generalizability of the present findings to real-world scenarios where a variety of food choices are available that could influence the quantities or types of beans consumed. However, other studies have also shown the efficacy of using biofortification to improve iron status in varying populations (31). Additionally, while the consumption patterns observed in the present study may not be generalizable to free-living conditions, the observed relation between iron status and physical performance should be generalizable to women who are iron depleted and have similar physical activity patterns (mostly sedentary with some light physical activity; data not shown) to that of the present study population.

The results of the present study suggest that even minor improvements in iron status can increase work efficiency. In addition, to our knowledge, this study is the first to demonstrate that changes in hemoglobin or ferritin from consuming relatively low doses of iron from biofortified and control beans over 18 wk can produce improvements in work efficiency in iron-deficient, untrained university women.

## Supplementary Material

nxaa016_Supplemental_File

## Notes

This study was funded by grants from HarvestPlus/International Food Policy Research Institute and Cornell University.

Author disclosures: JDH served as a paid technical consultant for HarvestPlus/International Food Policy Research Institute. LMP is currently employed by HarvestPlus/International Food Policy Research Institute. The other authors reported no conflicts of interest. The funders of this study had no role in the study design, implementation, data collection, data analysis, data interpretation, or writing of the manuscript.

Supplemental Table 1 is available from the “Supplementary data” link in the online posting of the article and from the same link in the online table of contents at https://academic.oup.com/jn/.

SVL is deceased.

Abbreviations used: AGP, α1-acid glycoprotein; Control-Bean, low-iron, conventional beans; CRP, C-reactive protein; EAR, Estimated Average Requirement; Fe-Bean, iron-biofortified beans; HR, heart rate; ITT, intent-to-treat; sTfR, soluble transferrin receptor; *V*O_2_, volume of oxygen consumed.
