# Supplementary material for: Increased Iron Status during a Feeding Trial of Iron-Biofortified Beans Increases Physical Work Efficiency in Rwandan Women
Source: J Nutr. 2020 Jan 31;150(5):1093–9. doi: 10.1093/jn/nxaa016 (PMC7198300; doi:10.1093/jn/nxaa016)
Supplement: nxaa016_Supplemental_File [file nxaa016_supplemental_file.docx]

**Title**: Increased iron status during a feeding trial of iron-biofortified beans increases physical work efficiency in Rwandan women

**First author**: Luna SV

**Online Supplemental Materials**

**
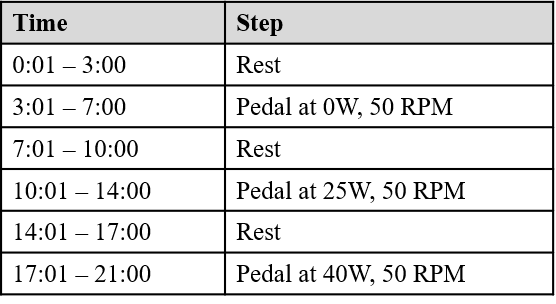
Supplemental Figure 1**: Protocol of the Graded Submaximal Exercise Test

Abbreviations: RPM – revolutions per minute; W – watts; HR – heart rate; BPM – beats per minute
